# Neurotological findings in patients with Parkinson's disease

**DOI:** 10.1016/S1808-8694(15)30567-X

**Published:** 2015-10-19

**Authors:** Jackeline Martins Bassetto, Bianca Simone Zeigelboim, Ari Leon Jurkiewicz, Karlin Fabianne Klagenberg

**Affiliations:** 1 MSc, Speech and Hearing Therapist.; 2 PhD, Coordinator of the MSc Program at Tuiuti University in Paraná.; 3 PhD, MD, Professor.; 4 MSc, Speech and Hearing Therapist. Tuiuti University in Paraná

**Keywords:** parkinson's disease, electronystagmography, vestibular function tests, dizziness

## Abstract

The number of elderly people is increasing considerably in our settings, and with that we have a matching increase in chronic-degenerative diseases - such as Parkinson's Disease (PD), which has balance-related symptoms associated and is increasingly more prevalent in the elderly population.

**Aim:**

Study labyrinth exams in PD patients and associate them with vestibular disorders. Study design: contemporary cross-sectional cohort.

**Materials and Methods:**

Thirty patients were assessed, twenty females and ten males, at the age range of 48 - 84 years. Procedures: anamnesis, ear inspection and vestibular assessment by means of a vecto-electronystagmography (VENG).

**Results:**

a) As to the neurotological complaints reported in the anamnesis, there was a prevalence of: tremor (100.0%), dizziness (43.3%), tinnitus (40.0%), gait unbalance and falls (36.6%) in each; b) in assessing the vestibular function, there was a significant difference in the rate of altered exams (p=0.0000); c) Most alterations happened in the peripheral vestibular system (93.3%) and the caloric test, with a predominance of bilateral labyrinth hyporreflexia (30,0%); d) The exam results were correlated with the vestibular symptoms and we noticed that there were no significant differences.

**Conclusion:**

this study showed us a significant number of altered exams, unrelated to symptoms.

## INTRODUCTION

The aging process manifests itself through the linear functional decay of various bodily organs as a function of time, while it is not possible to exactly define when the transition into it takes place, as in other stages. At the end of the third decade of life, anatomic and functional changes attributable to aging are more evident^1^.

The elderly population is growing considerably in Brazil due to the improvements in public health care and progress in medical science. Today, 15 million people are over the age of sixty; Brazil ranks sixth in the world in number of elderly citizens - 32 million - according to the World Health Organization. Estimates from the WHO indicate that there will be two billion people over the age of sixty in 2025^2^.

The boundary between adult and elderly age is 65 years for developed countries and 60 for developing nations. Such criterion is widely adopted by elderly care institutions^3^.

Chronic degenerative diseases such as Parkinson's and the cognitive alterations observed in Alzheimer's have seen increases in their incidence rates. Parkinson's is known to be one of the most frequent neurologic disorders, compromising mainly the motor system and with yet unknown causes^4^. It is known that there is a reduction in the production of dopamine by the substantia nigra^4^. With aging, the speed at which nerve impulses are transmitted is reduced and neurotransmitters are altered^5^. The lack of dopamine - a neurotransmitter that acts on the basal nuclei - leads to the onset of Parkinson's^5^. For some authors^6^ the etiology of the disease is obscure, but several factors such as genetics, atherosclerosis, excessive accumulation of oxygen free radicals, viral infections, head trauma, use of antipsychotic medication, and environmental factors may trigger the disease.

Several changes take place, among them the involvement of the central nervous system's ability to process vestibular, visual, and proprioceptive signals associated with maintaining bodily balance, diminishing the ability to modify adaptive reflexes^7^. Symptoms like dizziness and imbalance also occur as a consequence of such sensorial alterations, which may affect persons of other ages, although they are more frequently found in elders above 65 years of age^7^. Patients above the age of 75 have dizziness as the most evident symptom^8^, ^9^. Other symptoms may be present along with etiologic causes of sensorial nature, stroke, cardiovascular disorders, metabolic diseases, neck and neurologic alterations, bone and degenerative diseases, among others^8^, ^9^. Changes in postural control in elderly populations lead to increased risk of falling, its consequent sequelae and increased morbidity^9^, ^10^, ^11^.

Vestibular tests comprise a set of procedures that allows for the functional assessment of the systems associated with maintaining bodily balance, i.e., the triad that makes up the vestibular system^12^. The evidences raised by vestibular examination enable the assessment of the relationship between balance and function of the posterior labyrinth, vestibular branches of the eighth cranial nerve, vestibular nuclei on the floor of the fourth ventricle, vestibular pathways and, above all, the vestibulo-ocular, vestibulocerebellar, vestibulospinal, and vestibulo-proprioceptive cervical interrelations, in order to establish the site of injury^7^.

As longevity is extended and imbalance reduces the quality of life of Parkinson's patients, this paper aims to analyze the findings of inner ear examination and correlate them to the vestibular symptoms observed in this population.

## MATERIALS AND METHOD

This study was approved by the Institutional Ethics Committee under permit 008/2005 and duly authorized by the patients who signed a Free Informed Consent form.

Thirty patients with Parkinson's were included, being 20 males and 10 females with ages ranging between 48 and 84 years.

This is a cross-sectional study in which patients were assessed regardless of the type of treatment and time for which they had been treated.

The study comprised the following steps:

### Interview

Patients answered a questionnaire to assess their otoneurologic symptoms and signs. Patients with impeditive psychological, visual, and musculoskeletal impairment and other disorders that rendered vestibular testing impossible were excluded.

### Otorhinolaryngological evaluation

Tests were conducted to exclude alterations that could interfere with the examination.

### Vestibular testing

Patients underwent the following tests:

### Without recording

* Search for nystagmus and positional vertigo;
* Search for spontaneous and semi-spontaneous nystagmus with eyes opened, looking ahead, looking thirty degrees to the right, left, up, and down.

### With recording

VNG was carried out using a thermo-sensitive VN316 Berger device with three recording channels. After the skin in the periorbital region was cleaned with alcohol, an active electrode was fixated with electrolytic paste to the lateral angles and to the medial frontal line of the patient's eyes, thus forming an isosceles triangle to allow for the identification of horizontal, vertical, and oblique eye motion patterns. This type of VNG enables more accurate measurements of angular speed in the slow component (vestibular correction) of the nystagmus.

A Ferrante decreasing pendular rotary chair, an EV VEC Neurograff visual stimulator, and an NGR 05 Neurograff caloric testing device with air temperatures of 42°C, 18°C and 10°C, were used in the caloric tests.

The following eye and labyrinth tests were carried out during VNG, as described by Mangabeira-Albernaz et al.^13^.

* Eye motion calibration. In this stage of the examination we looked at tracing consistency to allow for comparisons against other research efforts;
* Search for spontaneous (open and closed eyes) and semi-spontaneous (open eyes) nystagmus. In this stage we looked at occurrence, direction, inhibitory effect of eye fixation and maximum speed of the slow component of ocular nystagmus;
* Search for pendular tracking, looking at occurrence and type of tracing;
* Search for optokinetic nystagmus, assessing occurrence, direction, slow component maximum speed in counterclockwise and clockwise eye motion, and directional preponderance;
* Search for pre and postrotational nystagmus using the decreasing pendular rotation test to stimulate the anterior and posterior lateral semicircular canals. Occurrence, direction, frequency on counterclockwise and clockwise rotation, and directional preponderance were observed;
* Search for pre and post caloric testing nystagmus through stimulation of the lateral semicircular canals. Air was injected in both ears in three sessions of 80 seconds each, at 42°C, 18°C and 10°C. Responses were recorded first with the patients closing their eyes and then with their eyes opened to look at inhibitory effect of eye fixation. In this test we looked at direction, absolute values for slow component maximum speed, directional preponderance, and post-caloric testing labyrinthine nystagmus prevalence.

### Statistical method

A descriptive analysis of the data collected from the otoneurologic interviews and vestibular tests was produced. Then the ratios of altered and normal test results were analyzed. Fischer's test was then applied to look at altered and normal vestibular test results with and without symptoms, and with rotational and non-rotational dizziness. After both tests were applied, a significance level of 0.05 (5%) was adopted to reject the null hypothesis.

## RESULTS

**Chart 1:**
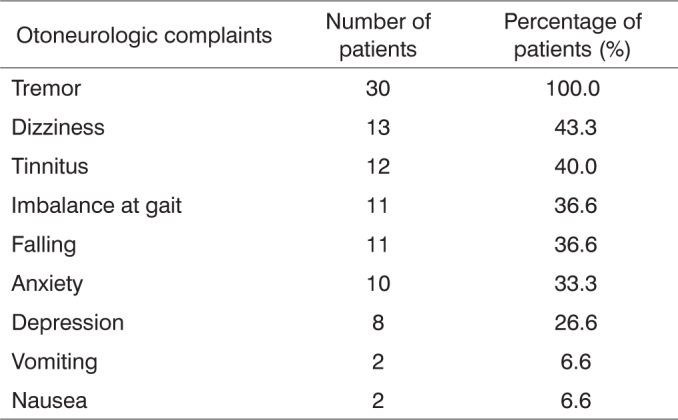
Otoneurologic complaints reported by thirty patients with Parkinson's disease.

The main otoneurologic complaints referred to during the interviews were trembling, dizziness, tinnitus, imbalance at gait, and falling, as shown in Chart 1.

Positional nystagmus testing could not be done in 27 patients, as they were physically unable to complete the test; only three patients was it done without occurrences of nystagmus and/or vertigo.

**Chart 2:**
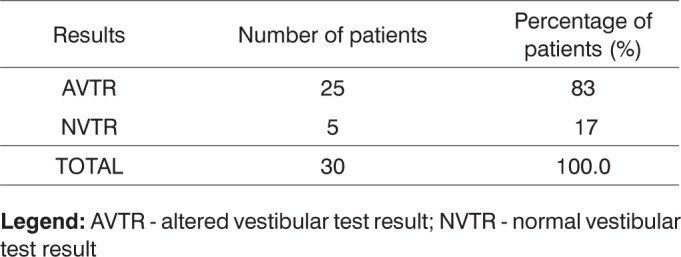
Vestibular test results for thirty patients with Parkinson's disease.

**Legend:** AVTR - altered vestibular test result; NVTR - normal vestibular test result

Vestibular testing showed altered results in 25 cases; 23 of which resided in the peripheral vestibular system and two in the central vestibular system, as shown in Chart 2.

After applying the ratios test and considering a significance level of 5% (α=0.05), a statistically significant difference was found between the ratio of altered and normal test results, as p=0.0000< (α=0.05).

**Chart 3:**
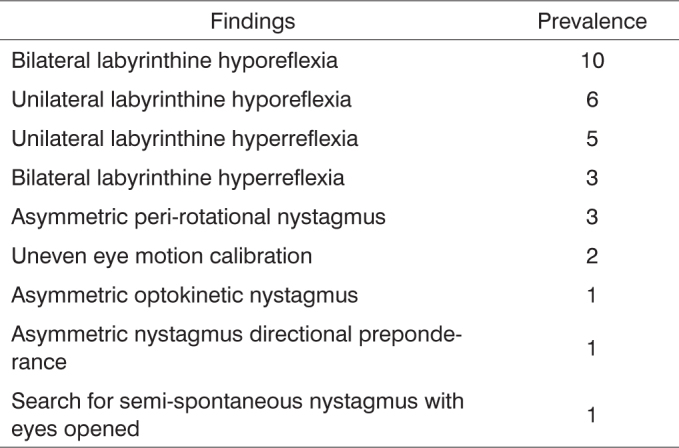
Prevalence of normal findings on the vestibular tests of thirty patients with Parkinson's disease.

Most vestibular altered results were found in the caloric testing; bilateral labyrinthine hyporeflexia was the most prevalent finding, as shown in Chart 3.

**Chart 4:**
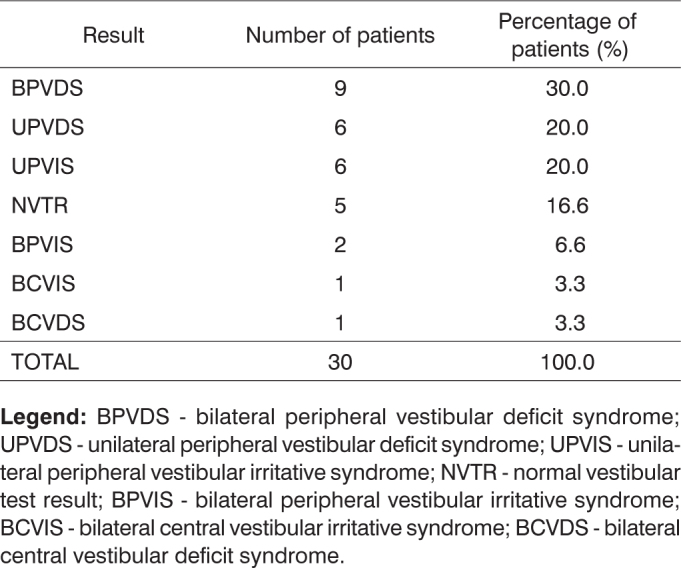
Vestibular test results for thirty patients with Parkinson's disease.

**Legend:** BPVDS - bilateral peripheral vestibular deficit syndrome; UPVDS - unilateral peripheral vestibular deficit syndrome; UPVIS - unilateral peripheral vestibular irritative syndrome; NVTR - normal vestibular test result; BPVIS - bilateral peripheral vestibular irritative syndrome; BCVIS - bilateral central vestibular irritative syndrome; BCVDS - bilateral central vestibular deficit syndrome.

Vestibular examination identified nine cases of bilateral peripheral vestibular deficit syndrome; six cases of unilateral peripheral vestibular deficit syndrome; six cases of unilateral peripheral vestibular irritative syndrome; five cases of normal vestibular findings; two cases of bilateral peripheral vestibular irritative syndrome; one case of bilateral central vestibular irritative syndrome; and one case of bilateral central vestibular deficit syndrome, as shown in Chart 4.

**Chart 5:**
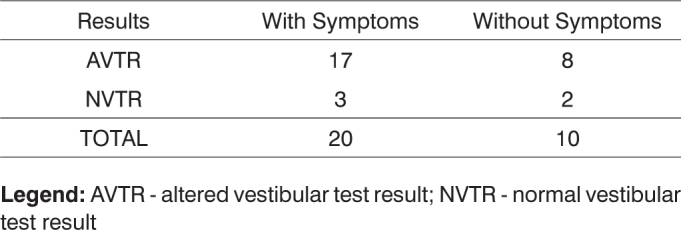
Correlation between vestibular test results and presence of vestibular symptoms in thirty patients with Parkinson's disease.

**Legend:** AVTR - altered vestibular test result; NVTR - normal vestibular test result

**Chart 6:**
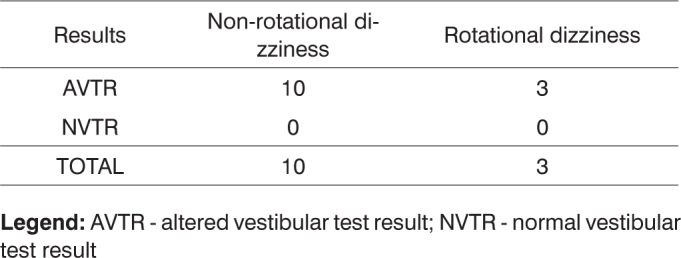
Correlation between vestibular test results and dizziness in thirty patients with Parkinson's disease.

**Legend:** AVTR - altered vestibular test result; NVTR - normal vestibular test result

When correlating test results to vestibular symptoms referred to by the patients and to presence or absence of rotational and non-rotational dizziness, we identified 17 cases of altered results with symptoms and eight cases of altered results without symptoms. From the five patients with normal vestibular test results, three had vestibular symptoms and two were asymptomatic. From the 25 patients with altered vestibular test results, ten had non-rotational dizziness and three had rotational dizziness. There was no correlation between rotational and non-rotational dizziness among the five patients with normal test results, as shown in Charts 5 and 6.

After applying Fischer's test and considering a significance level of 5% (α=0.05), no statistically significant difference was found between the ratios of patients with altered and normal test results and those with and without symptoms, as p=1.0000> (α=0.05).

After applying Fischer's test and considering a significance level of 5% (α=0.05), no statistically significant difference was found between the ratios of patients with altered and normal test results and those with and without rotational dizziness, as p=0.5280> (α=0.05).

## DISCUSSION

When looking at interview findings, trembling, dizziness, tinnitus, imbalance at gait, and falling were among the most prevalent complaints. According to Smeltzer and Bare^6^ and Reichert et al.,^14^, trembling is the early manifestation of the disease and is followed by stiffness and bradykinesia. In his studies, Whitney^15^ mentions dizziness as one of the most frequent complaints. Jones and Godwin-Austen^16^ state that gray matter central nuclear masses hold practically all the dopamine present in the human brain. Dopamine is an amine neurotransmitter and the lack thereof results in neuronal degeneration. Phenothiazines chemically block its mode of action. The mechanisms by which such neurochemical alteration triggers the symptoms of the disease still remain obscure.

It should be considered that the aging of sensorial systems, mainly those connected to eyesight, proprioception, plantar pressure receptors, and inner ear function, leads to neuronal losses in all levels, starting from the sixth decade of life and growing in intensity as of seventy years of age (Mota et al.)^17^. Conscious and unconscious proprioceptive impulses arising from the periosteum, joint capsules, ligaments, striated muscle, fascia, and aponeurosis allow the cerebral and cerebellar cortices and the subcortical centers to provide for muscle tone, balance, and motor coordination.

Altered vestibular test results were seen in the peripheral vestibular system of 23 patients and in the central vestibular system of two patients. The higher prevalence verified in the peripheral vestibular system has also been elicited in several trials comprising older populations, as seen in Mota et al.,^17^ and Gushikem^18^. Greater numbers of central vestibular syndromes in Parkinson's patients were not observed due to the development stage of the disease encountered in our group of patients.

As to abnormal VNG findings, we observed uneven eye motion calibration, semi-spontaneous nystagmus (with eyes opened), asymmetric optokinetic nystagmus, asymmetric peri-rotational nystagmus, asymmetric nystagmus directional preponderance, unilateral and bilateral labyrinthine hyporeflexia and hyperreflexia. Bilateral labyrinthine hyporeflexia was also noted as a significant finding by Reichert et al.^14^ and Gushikem^18^ in Parkinson's studies with older patients. Dolowitz^19^ found hyperreflexia in most analyzed patients. According to Silveira et al.^20^ reduced response in caloric tests may occur due to aging-related alterations in the vestibular system. Various studies make reference to loss of hair cells in the cristae ampullaris and maculae, reduced number of nerve cells in the vestibular ganglion, degenerated otocones, reduced labyrinthine blood flow, progressive neural stability depression, reduced compensatory capacity of ocular-vestibular reflexes (responsible for maintaining stable eyesight during head movements) and vestibular-spinal reflexes (responsible for bodily balance), all contributing to reduced velocity of eye tracking motion and rotational and caloric hyporeactivity of both peripheral and central vestibular systems (Whitney,^15^; Silveira et al.^20^ and Hain et al.^21^).

Higher prevalence rates of peripheral vestibular deficit syndrome were found in relation to peripheral vestibular irritative syndrome, as was the case in the studies conducted by Gushikem^18^. Mota et al.^17^ reported more cases of peripheral vestibular irritative syndrome instead. Fukuda et al.^22^ identified both deficit and irritative syndromes.

The correlation between vestibular symptoms and presence of rotational and non-rotational dizziness in relation to altered and normal vestibular test results pointed to higher prevalence of vestibular symptoms in patients with altered test results, as also found by Reichert et al.^14^. The incidence of non-rotational dizziness was also higher among patients with altered vestibular test results. Whitney^15^ estimated that most cases of dizziness stem from vestibular disorders. According to Ganança and Caovilla^23^ the main symptom arising from vestibular disorders in elderly populations is rotational dizziness.

## CONCLUSION

This study allowed the verification of a significant number of altered test results in symptom-free subjects, stressing the importance of carefully assessing Parkinson's patients so as to better understand how the disease affects their balance system and thus aid in the design of their speech and hearing therapies.
